# Landscaping the early child care policy architecture in sub-Saharan Africa and an in-depth review of Kenya

**DOI:** 10.3389/fpubh.2026.1700472

**Published:** 2026-05-21

**Authors:** Margaret Nampijja, Patricia Kitsao-Wekulo, Linda Oloo, Ruth Muendo, Hilda Owii, Gloria Langat

**Affiliations:** 1 Research Division, African Population and Health Research Center, Nairobi, Kenya; 2 School for Policy Studies, University of Bristol, Bristol, United Kingdom

**Keywords:** 0–3 years, early child care, gaps, policy frameworks, policy recommendations

## Abstract

**Background:**

The demand for quality early child care is rapidly rising globally, yet in sub–Saharan Africa (SSA), there is limited evidence on what drives quality, provision, and distribution of the burden of care across the different providers and what works locally. In particular, it is not clear to what extent existing local and regional as well as global policies and regulatory frameworks set the agenda and priorities for child care, and how explicit they are for care of the 0–3 year old children (early child care). Within a larger project that sought to explore the dynamics of the care economy in the Kenyan context, we conducted a review of the policy architecture of existing national, regional and global policies on early child care to evaluate the scope, implementation strategies, budget commitment and any gaps and challenges in the scope and implementation. We sought to understand the extent to which these policies cater for early child care in non-health care environments and identify gaps particularly in the distribution of the burden (responsibility) across different players at various levels. In this study, the term child care is used to refer to care of young children within their family environment and in any external non-clinical environments, and employs the holistic view of care across the five domains of the nurturing care framework (good health, adequate nutrition, responsive caregiving, opportunities for early learning, safety, and protection) (WHO, 2017). We focused on early child care i.e., care for children ages 0–3 years. Because of its comprehensive nature, early child care is therefore a heavy responsibility but which most of the time disproportionately falls on women.

**Methodology:**

A qualitative review of published policy documents both peer reviewed and gray literature was conducted. Using an internally developed review protocol, we searched and reviewed global, regional (SSA) and national policy documents on early child care, focusing on the five areas of the nurturing care framework which include good health, nutrition responsive caregiving, opportunities for early learning, safety and security. For each document, we evaluated the extent each provides for children's rights and wellbeing as stipulated in the UN Convention on the Rights of the Child, strengths and gaps, and made recommendations for improvement.

**Findings:**

Findings reveal significant progress in the care policy landscape addressing the various aspects of nurturing care. However, a lot remains to be done. Lack of clarity of policies on early child care for children aged 0–3 years; weak linkages between policy frameworks and implementation; and, the roles of various players. The need for resources to support implementation of the policies especially in resource limited communities as well as a system for regular tracking and evaluation of policy implementation and impact against the intended outcome indicators was evident.

**Conclusion:**

Collaborative efforts from policy actors are needed to address the policy gaps toward optimum care for the wellbeing of all children in Africa.

## Background

Early child care, defined as the provision of children's needs across the five domains of the nurturing care work within and outside of their family environment is critical for the wellbeing and development of children. Since the launch of the Nurturing Care Framework (NCF) by WHO in 2018, there has been a growing interest and strategic efforts to optimize care including instituting policy frameworks to ensure that children receive the necessary care.

In Africa however, early child care (ECC) (care for children below the age of 4 years) remains mostly under the remit of informal caregivers with almost no public care support, provisioning or social infrastructure (1–4). The demand and need for care is set to rise drastically in the coming decades due to the demographic and epidemiological trends (UNDESA-PD, 2019). Informal ECC provision without public support or care infrastructure is often associated with poor quality care, work-family tensions, time constraints and poor health and mental wellbeing for both the children and caregiver (1). Many informal caregivers are women, impacting their social and economic opportunities and hence a major driver of gender inequality (5). Social inequities and care quality deficits disproportionately affect the poor and those in precarious economic circumstances (1, 6).

The Care Economy Africa (CEA) project, implemented in Kenya and Senegal, sought to advance and inform policy action to develop transformative care systems and economies that build on existing African values and strengths to enhance care quality for young children in need of, and, receiving ECC. The project envisaged a transformative care system that is responsive to people's needs, values and priorities and that contributes to achieving individuals', communities' and national aspirations for future progress. The values, knowledge and priorities of those giving and receiving care or with a future stake in care in the African contexts, have not been sufficiently considered, let alone centered, in the articulation of the key “problematics” of care and the policy solutions needed to address them. The overarching goal of the project was to understand the relevance of the current global women unpaid care work and women economic empowerment (WUCW/WEE) agendas and underlying gender perspectives for shaping care futures in Africa—including how they drive the current inequalities in child care. Why is early child care important and to what extent is it grounded in current policies?

Quality child care is defined as a safe, nurturing, and stimulating environment where warm, responsive adults foster children's physical, social, emotional, and cognitive growth through age-appropriate activities and strong family partnerships, ensuring readiness for school and life. It has the potential to provide multiple social and economic benefits for the child, their families and societies (7, 8). High quality child care gives children the best start to life and increases their life chances (9, 10) and it addresses Sustainable Development Goal (SDG) 3 and 4. Quality child care means that the child is safe and healthy and accessing opportunities for learning (SDG 3 and 4). It has been linked to specific outcomes including foundational literacy, numeracy and language skills, and later academic outcomes (SDG 4). For caregivers (usually women), child care support enables women's participation in the labor force (11–13) thereby contributing to SGD 1 and 8. Access to quality child care enables parents (women) to pursue their careers but also allows parents to take time out for their own health (SDG 3). Mothers have peace of mind at work, resulting in more efficiency and productivity (SDG 1 and 8). Child care providers (mostly women) earn a living from the services (SDG 1 and 8). Improved parents' income contributes to better family and national economic growth (SDG 1 and 8). Benefits of child care align with SDGs, and to encourage greater investment in childcare, it's important to highlight the strong evidence showing that supporting children in their early years can significantly reduce health disparities and contribute to personal, social, and economic growth (4, 9, 10, 14). Despite the recognized importance of childcare, 350 million children do not access quality childcare, contributing to the 250 million worldwide that may not achieve their developmental potential. This is in part due to system challenges, including lack of policies and regulations on child care or poor implementation of the guidelines at different levels. Policies have a critical role in influencing access and practices in child care. Non-inclusive policies that do not cater for the diverse population needs can underlie the inequalities in the quality and access to childcare services. Studies have demonstrated that comprehensive and well-articulated policies in child care translate to quality child care practices that consider all the key aspects of the child's wellbeing (15). In sub-Saharan Africa, there are several local, regional and global policies, that may have a bearing on the current inequitable access to child care support, and unequal distribution of the burden to women; however, the extent of these disparities is yet to be examined.

It is on this background, and in line with the objectives of the main study, that we undertook a comprehensive review of the policy architecture of existing local, regional, and global policies on early child care to evaluate the scope, implementation strategies, budget commitment, and any gaps and challenges that need to be addressed. We aimed to examine the extent to which the policies cater for ECC in non-healthcare environments and identify gaps that exist in these policies particularly in distribution of the burden across different players at different levels. The focus on non-health care environments for the review was informed by the objectives of the main study of which the review was part. Within the main study, we aimed to map and profile the state of care economy including at policy level to inform workable approaches to recognition and redistribution of care in the African context. Whereas the health sector provides clear guidelines for the healthcare of populations including young children and old people, policies on other aspects of care are not clear. This review therefore critically examined existing local, regional and global policy frameworks that provide for early child care uncovering their strengths and aspects that that need to be strengthened. The review provides recommendations for strengthening policies including for improving clarity, implementation, resourcing and accountability. To our knowledge, this is the first policy review to comprehensively evaluate policies that provide for child care in the African context. Previous reviews were generally limited to local policy frameworks and regulations. For instance, a review by Adebiyi et al. (16) examined policies that provided for Fetal Alcohol Spectrum Disorder considered in South Africa, and covered the broad aspects of the nurturing care framework. However, the review was limited to local (South African) policies despite that local policies are often linked to regional and global policies and hence a gap in local policy might be a reflection of similar gaps in the regional or global policy frameworks.

## Methods

### Study design

Within a study aimed at understanding the dynamics of care economy in the Kenyan context, we conducted a review to understand the policies that speak to early child care in Kenya, sub-Saharan Africa and at the global level. This part of the study was focused on a review of available policy documents using a qualitative approach. Relevant peer reviewed and gray literature was reviewed. The policy review was guided by a methodological framework proposed by Askey and O'Malley (17) and used a sequential process including (i) definition of the research questions, and scope, (ii) identification and generation of the relevant policy documents from different databases, (iii) screening and selection of eligible documents, (iv) data extraction, and (v) collating and summarizing the results. The search strategy was based on the WHO's 2017 Nurturing Care Framework (NCF) which demonstrates the interconnected roles of various sectors such as nutrition, health, education, finance, social protection, labor, water and sanitation, and gender in promoting a child's wellbeing. The NCF was deemed ideal as it provides a holistic approach to children's developmental needs that are critical especially during the early years from birth to age three.

### Scope and key questions

We reviewed local (Kenya) legal frameworks on ECC, as well as sub-regional in East Africa, regional Africa, and global policies. For each document, we evaluated the extent each provides for children's rights and wellbeing as stipulated in the UN Convention on the Rights of the Child, plus the strengths and weaknesses or gaps, and based on this we made recommendations for improvement. Children's rights is defined as the fundamental human rights and entitlements for individuals under 18, ensuring survival, development, protection, and participation. As per the UN Convention on the Rights of the Child, these rights include education, health, nutrition, shelter, protection from abuse, and freedom of expression. These rights are legally binding. For young children (0–3 years), these rights align with the nurturing care framework, which is the backbone for quality early child care.

The rationale for focusing on Kenya was based on the objectives of the main study, which aimed to understand the drivers of care economy in Kenya, in order to make recommendations for recognition and redistributions of care in the Kenyan, context. There may be similarities in ECC policy frameworks across the sub-Saharan African region particularly in regards to the shared (regional) policies, from which National policies are drawn. Hence, findings from Kenya can be generalized to contexts beyond Kenya. On the other hand, specific aspects including subnational level frameworks and implementation approaches that are unique to Kenya may not be generalizable to other contexts.

The review focused on the following specific questions:

a) What definitions of ECC are used in instruments, if any? Are there agreements in definition among national (and subnational) instruments; and between national, regional and continental instruments? What terms are used for informal/family and formal/organized care?
b) What kinds of instruments (legal, policy; sector/cross-cutting) speak directly to ECC at global, continental, regional, national, and sub-national levels?
c) What are the ‘problems' in ECC that the policies aim to address and what overall aims are they are pursuing? What is the relative focus on—the rights and wellbeing of the care recipient and caregiver? To what extent do national instruments make reference to or align with continental or global frameworks?
d) What are the specific national/subnational policy directions in relation to:
  - The relative responsibility of family and state (and others) regarding the provision of ECC?
  - The role of “formal” care service provision vs. care provision by family members/domestic workers; and the kinds of formal services considered?
  - The required quality of ECC?
  - Support for family carers?
  - Support for domestic workers providing care?
  - Expansion of/provision of formal ECC services and the kinds of formal services proposed?

The rationale for profiling the policies along these questions was not only to understand their scope but also to examine the extent they influence the dynamics in early child care economy including access, quality, prioritization, and roles and responsibilities and ultimately how they can be leveraged to reduce the existing inequalities in child care in the African context. To the extent possible and as much as was relevant to the policy in question, the different policy documents were assessed along each of these questions.

### Search strategy

In developing our search strategy, we drew inspiration from the Nurturing Care Framework (NCF). The NCF emphasizes the interconnected roles of various sectors such as nutrition, health, education, finance, social protection, labor, water and sanitation, and gender in promoting a child's wellbeing. The NCF, introduced by WHO in 2018, takes a well-rounded approach to nurturing children's development, especially during the crucial early years from birth to age three. It emphasizes the vital role of creating a safe, supportive and nurturing environment where young children can thrive, with access to proper nutrition and responsive care. Given the close link between women's roles and child care, we also broadened our search to include policies related to women's economic empowerment (WEE), recognizing its significant influence on caregiving practices and overall child development.

### Identifying relevant studies and screening them for eligibility

The study team searched for both peer-reviewed literature and gray literature from 2010 to 2022 (when the review was done). The rationale for starting at 2010 was based on the period when the Kenyan 2010 constitution, which established county governments, was promulgated. Databases searched for peer reviewed literature included: PubMed, ScienceDirect, Open Gray, African Journals Online (AJOL), Scopus, APA PsycINFO, or PsycNet. For the gray literature search we used online resources such as Google Scholar as a search engine and global, regional, national and sub-national websites/reports. Search terms shown in Table 1 were used to find documents from the databases and gray literature. The databases were distributed among the six members of the team. When all the policies were generated, they were screened for duplicates and eligibility by the team working in pairs. Any discrepancy between the pair was resolved by a third person. The eligible policy documents were distributed among the team who read and extracted and synthesized the information using the review questions listed above. The information was consolidated and further synthesized by the lead author. Updated versions were then reviewed and edited by the rest of the co-authors.

**Table 1:** The search terms used to find relevant literature.

Region	Search terms
GLOBAL	[(“Childcare” OR “infant care” OR “care for children” OR “ECC”) AND (“health^*^” OR “early learning” OR “safety” OR “security” OR “protection” OR “stimulation” OR “care^*^” OR “nutrition” OR “early learning” OR “sanitation” AND (“policy” OR “legal framework” OR “guidelines”) AND (“international” OR “Global”)]
SSA	(“Childcare” OR “infant care” OR “care for children” OR “ECC”) AND (“health^*^” OR “early learning” OR “safety” OR “security” OR “protection” OR “stimulation” OR “nutrition” OR “early learning” OR “sanitation” AND (“policy” OR “legal framework” OR “guidelines”) AND (“SSA” OR “sub-Saharan Africa”)]
Kenya	(“Childcare” OR “infant care” OR “care for children” OR “ECC”) AND (“health^*^” OR “early learning” OR “safety” OR “security” OR “protection” OR “stimulation” OR “nutrition” OR “early learning” OR “sanitation” AND (“policy” OR “legal framework” OR “guidelines”) AND (“Kenya”)]
County level frameworks	(“Childcare” OR “infant care” OR “care for children” OR “ECC”) AND (“health^*^” OR “early learning” OR “safety” OR “security” OR “stimulation” OR “nutrition” OR “early learning” OR “sanitation” AND (“policy” OR “legal framework” OR “guidelines”) AND (“County Name”)]

### Inclusion and exclusion criteria

We focused on global policies, regional policies (East Africa and sub-Saharan Africa), national policies (Kenya), and subnational guidelines including the Kenya County Integrated Development Plans (CIDPs) (29). We restricted our focus on early care for children aged 0–6 years; and the 5 domains of the Nurturing Care Framework. Within each policy we also examined the role of different players in supporting the implementation. We reviewed documents published from 2010 to 2022. To ensure that the documents included were quality and authentic, we used documents published on official websites of the different institutions, and the current (most updated) and approved versions of the documents. Draft versions were not included.

### Document review and analysis

We used an internally standardized data extraction form and extracted data including policy name, date of publication, and level of policy (subnational, national, regional, continental, and global). These variables were used to describe the policy documents. The analysis of each policy document was aligned with the key review questions listed above. Information from the different policies was combined and a discussion of the synthesized perspectives generated. Six members of the research team participated in the entire policy review process.

## Results

A summary description of the documents retrieved and included in the review is presented in Table 2. Altogether 69 documents were reviewed including three global policy frameworks, nine regional level documents, four national (Kenya) level policies, and 52 county level documents (five child care Acts and 47 County Integrated Development plans).

**Table 2:** List of policy frameworks reviewed.

Category of policy document	Name of policy document
Global policy frameworks	
	The United Nations Convention on the Rights of Children (UN CRC)
	The World Bank Agenda on Gender
	Sustainable Development Goals
Regional policy frameworks	The United Nations Economic Commission for Africa (UN ECA)
	The African Union (AU)
	The African Charter on the Rights and Welfare of the Child (ACRWC)
	The African Development Bank (AfDB)
	Southern African Development Community (SADC)
	Economic Community of West African States (ECOWAS)
	The Common Market for Eastern and Southern Africa (COMESA) Treaty
	The Economic Community of Central Africa States (ECCAS)
	The East African Community
Local (Kenya) policy frameworks	
National policy frameworks	The Children's Act (2001, revised 2022)
	National Early Childhood Development Framework of 2006
County level child care facilities Acts and Plans	Nairobi City County Childcare Facilities Act (2017)
	The Embu County Childcare Facilities Act, 2016
	The Mombasa County Childcare Act; Act, 2016
	The Mandera County Orphanage Facilities Act, 2021
	The Nakuru County Early Childhood Education Act, 2016
	The Tana River County Early Childhood Education Act, 2016
	The 47 County Integrated Development Plans (CIDPs); one for each County

### Definitions of terminology around early child care

Many of the regional documents do not include an operational definition of ECC but they recognize the need to promote the rights and welfare of young children as espoused in the UN Convention on the Rights of the Child (CRC) (1989), the African Charter on the Rights and Welfare of the Child (ACRWC) (1990; became effective in 1999).

In Kenya, since 2010, ECD including child care has been a devolved function of the Counties. This means that many of the decisions made are at the County level. A few of the operations for example the National ECD policy, were retained at the National level. At national level, the various documents provide definitions of child care and related terms such as caregiver, child care provider, care center, orphanage facilities and early childhood services. According to the Nairobi City County Childcare Facilities Act of 2017, a caregiver is described as someone responsible for looking after children within a childcare facility. The Act defines childcare as the temporary supervision and care of a child in a setting that is not the caregiver's primary home or personal living space. This care goes beyond basic supervision—it also includes the child's overall welfare, protection, guidance, and developmental support. A “childcare provider” is defined as “a person, their agents or representatives licensed or required to be licensed under the Act in order to establish, conduct or maintain a childcare facility.” The Care and Protection of Child Parents Bill (18) defines “childcare” as “services that have as their primary purpose the care and supervision of children.” In addition, a “care center” is defined as “a facility that provides child care services but does not include a family home.” Whereas these two instruments highlight the importance of supervision of young children as one component of care, they both stress that this care is provided outside the child's home. The Mandera County Orphanage Facilities Act (19) defines “orphanage facilities as any facility in the county, either home-based or institution-based where childcare or daycare is offered at any time to more than 10 children.” According to the Nakuru County Early Childhood Education Act of 2016 (20), “early childhood service” encompasses various forms of care and education settings—including early childhood education and care centers, home-based programs, and even hospital-based services. The Act further clarifies that an early childhood education and care center refers to any premises regularly used to educate three or more children under the age of six, whether for part of the day or a full day, for a duration not exceeding seven consecutive days.

## Landscape of the ECC policy architecture

### Global policy WHO nurturing care framework

Global policies generally focus on major, high level policy thrusts aimed at improving the environment in which children live so that they thrive and achieve their full potential. Occasionally, global policies directly address specific issues of early child care.

#### The United Nations Convention on the Rights of Children

The UN Convention on the Rights of Children (1989) commits States to protect the rights of children who are not able to access parenting to ensure that they receive good care. In December 2019, the UN General Assembly adopted a significant Resolution on the Rights of the Child (27), with a particular focus on children without parental care. The Resolution underscores the fundamental importance of children growing up in a supportive family environment and affirms every child's right to family life. It also brings attention to the specific needs and rights of children with disabilities within the context of family care. The Resolution strongly discourages the unnecessary separation of children from their families and condemns the arbitrary or unlawful deprivation of their liberty. It promotes efforts to reunite families when such reunification serves the best interests of the child. Importantly, it makes clear that children should never be removed from their families solely because of poverty or limited access to resources. Instead, it calls for strengthened support systems to help families stay together whenever possible.

In the context of early childhood care (ECC), the Resolution encourages governments to enhance child welfare and protection systems, with a particular focus on improving care reform efforts. It draws attention to the rising concerns surrounding the growing number of migrant children, particularly those who are unaccompanied or have been separated from their parents or primary caregivers; and emphasizes the need for diverse and appropriate alternative care options to ensure these children are protected. Additionally, the Resolution calls on States to take concrete steps to prevent the trafficking and exploitation of children, particularly those in institutional care or living without parental support. This includes addressing the risks associated with certain volunteer programs, such as those in orphanages linked to tourism, which can unintentionally contribute to exploitation and trafficking.

It's worth noting that the 2019 UN Resolution is silent with regards to the *responsibility* of providing child care and protecting children's rights. This omission might suggest the default gendered stereotypes in these considerations, and the assumption that it is women's responsibility. We can assume this arises from the lack of recognition of the unequal distribution of care and speculate that they leave the specification to the lower-level decision makers (States) to deal with.

#### The World Bank Agenda on gender

The World Bank Agenda on gender seeks to address the challenges associated with child care for women living in diverse circumstances. The goal of this is to protect children's rights and wellbeing, but also to promote equal opportunities for women to actively contribute to and benefit from the economic growth of society.

#### Sustainable Development Goals

Sustainable Development Goal (SDG) 4 “*Inclusive and equitable quality education and promote lifelong learning opportunities for all*” directly addresses early child care, and it emphasizes the need for responsive care and parenting for early learning, and highlights care areas that should be addressed, and that these should be specified in the national policies and subnational guidelines for implementation.

Overall, global policy frameworks provide guidance to promote the health and wellbeing of all children. These high-level policies serve as a reference for regional and national policies and guidelines that will enable implementation toward achieving the global targets.

### Regional policies and frameworks

#### The United Nations Economic Commission for Africa

The United Nations Economic Commission for Africa (ECA) (37) recognizes that advancing social development is key to driving structural transformation across the continent. Through its Social Development Policy Division (SDPD), ECA conducts research, offers technical support, and builds the capacity of member states to address critical social issues. These efforts span across employment creation, social protection, urban growth, migration, population dynamics, and the empowerment of women—all aimed at reducing social and gender disparities aligning with both regional and global commitments.

The SDPD also plays an important role in monitoring and evaluating international frameworks such as the International Conference on Population and Development (ICPD), the 2030 Agenda for Sustainable Development, the Beijing Platform for Action, and the Habitat Agenda. At the regional level, its work aligns closely with the African Union's Agenda 2063, which emphasizes the importance of human, social, and spatial development. The division's work is structured around four key areas: Employment and Social Protection, Urbanization, Population and Youth, and the African Centre for Gender (ACG). The ACG specifically supports governments in addressing gender inequality by offering technical tools, generating evidence for policymaking, and tracking progress toward gender-related commitments. Some of its key initiatives include conducting time-use surveys, gender-focused macroeconomic analyses, and strengthening gender statistics to better inform policies and programs.

#### The African Union

The AU, through its Continental Education Strategy for Africa (CESA 2016–2025), acknowledges how vital the early years are for a child's overall development. While the strategy primarily focuses on education, it also highlights the need for comprehensive approaches that support a child's first 1,000 days of life—widely recognized as a critical period for laying the foundation for lifelong learning and wellbeing. CESA encourages member states to take a holistic view of child development and strengthen collaboration across multiple sectors at both national and regional levels. It also emphasizes the positioning of parents and caregivers as a child's first educators. In addition, the AU calls on countries to invest in strong political leadership and advocacy to support effective policy development, implementation, and evaluation around early childhood development (ECD).

The Strategy emphasizes the importance of deliberate efforts to expand access to quality services, especially for children affected by poverty, illness, conflict, and those with special needs. It encourages Member States to advocate for and build the capacity of professionals to use child-centered, developmentally appropriate approaches—such as learning through play. It also underscores the need to improve working conditions for the early childhood education and development (ECED) workforce. In addition, the Strategy highlights the mandate of governments in promoting the use of locally available, low-cost materials for developing play and learning resources. A key priority is advocacy for increased funding and more effective resource allocation to ensure the long-term sustainability of ECED programs. To further strengthen early learning efforts, the Strategy calls on Member States to assess ongoing ECED initiatives, identify and scale up successful practices, and foster collaboration with key development partners such as the African Development Bank (AfDB), the UK's Department for International Development (DfID), the European Union (EU), the Global Partnership for Education (GPE), and the World Bank.

#### The African Charter on the Rights and Welfare of the Child

The African Charter on the Rights and Welfare of the Child (ACRWC) was adopted during the 26th Ordinary Session of the Assembly of Heads of State and Government of the African Union in Addis Ababa, Ethiopia, in July 1990 (36). It officially came into effect on November 29, 1999. The Charter outlines a broad range of rights for children across the continent. Of particular relevance to this discussion is Article 19, which emphasizes a child's right to parental care and protection. It affirms that every child has the right to be cared for by their parents and, whenever possible, to live with them. Separation from parents should only occur when a judicial authority determines under applicable law that it is in the best interest of the child.

The African Charter on the Rights and Welfare of the Child requires all member states to actively monitor and report on their progress in implementing the Charter's provisions. Kenya is among the 50 countries that have adopted and ratified the Charter. According to Article 43, each country is expected to submit a detailed report to the Committee through the Secretary-General of the African Union. This report should outline the measures taken to fulfill the Charter's obligations and highlight the progress made in realizing children's rights. The reporting process is structured in two phases: an initial report must be submitted within 2 years after the Charter comes into force for a given country, followed by subsequent reports every 3 years. These reports should provide a clear and comprehensive overview of how the Charter is being implemented, as well as identify any challenges or barriers encountered along the way. Importantly, once a country has submitted a thorough first report, it is not required to repeat the same foundational information in future reports. Instead, the focus should shift to updates, progress, and any new developments.

#### The African Development Bank

The African Development Bank (AfDB) (32) Group is committed to driving sustainable economic growth and social advancement across its regional member countries, with a central focus on reducing poverty. To support this mission, the Bank mobilizes and channels financial resources into strategic investments and provides policy guidance and technical support to help foster development. Aligned with the global Sustainable Development Goals, the AfDB is working toward ambitious targets aimed at unlocking the full human and economic potential of the African continent. As part of this effort, the Bank joined forces with other organizations through the Banking on Nutrition Partnership. This initiative was established in response to the persistent challenge of malnutrition, which continues to limit the physical and cognitive development of generations of Africans—ultimately affecting both health outcomes and long-term economic progress.

The Banking on Nutrition Partnership emphasizes investment in what it calls “Gray Matter Infrastructure”—a strategic focus on brain development and nutrition, especially in early life. Encouragingly, the initiative is already showing impact: at least 20% of its project interventions specifically target improvements in the health of women and children, a key demographic for driving lasting positive change.

#### Southern African Development Community

The Consolidated Treaty of the Southern African Development Community (SADC) outlines a dedicated commitment to supporting orphans, vulnerable children, and youth by addressing their fundamental human needs (34). This includes access to education and vocational training, healthcare and proper sanitation, food security and nutrition, child and youth protection, mental and emotional wellbeing, as well as broader social protection measures. However, there is no mention of ECC and there is no age category indicated for orphans and vulnerable children.

#### Economic Community of West African States

The Economic Community of West African States (ECOWAS) (33) has developed a Child Rights Policy that sets out a clear framework for protecting and promoting the rights of children across the region. This policy emphasizes several key areas essential to child survival and development. First, the right to life, which encompasses access to necessities such as proper nutrition, safe shelter, a decent standard of living, and quality healthcare services. These are the foundational elements every child needs to grow and thrive. The policy also recognizes every child's right to education and the importance of play, leisure, cultural engagement, and access to information. It supports the freedom of thought, conscience, and religion, nurturing the intellectual and emotional wellbeing of children. Another critical focus is protection from harm. The policy outlines safeguards against all forms of abuse, neglect, and exploitation. It also ensures the rights of children who come into contact with the legal system, protects children in the workplace, and provides support and rehabilitation for those who have experienced violence or mistreatment. Finally, the policy upholds children's right to participate. As children grow, they should have the freedom to express their views, be involved in decisions that affect their lives, join associations, and take part in peaceful assemblies. Encouraging their active participation helps prepare them to engage meaningfully in society as adults. Although not directly outlined, the document suggests the need to ensure that young children receive the best care possible so that their rights are protected.

### The Common Market for Eastern and Southern Africa treaty

While the Treaty of the Common Market for Eastern and Southern Africa (COMESA) (30) does not specifically mention Early Childhood Care (ECC) in its programs, related themes are addressed in the COMESA Social Charter (35). This Charter outlines 11 strategic pillars that guide the region's social development agenda. Among these are key areas such as employment and working conditions, labor laws, and child wellbeing. The core objective of the Social Charter is to ensure that all individuals in the COMESA region can live with dignity and reach their full potential. Although child care is not explicitly detailed, the Charter emphasizes the importance of the family as the foundational unit of society. It calls on Member States to strengthen families and recognize their vital role in social development. This includes acknowledging the rights, roles, and responsibilities of all family members, particularly children, the older people, and people with disabilities.

Furthermore, the Charter urges Member States to uphold and promote universal human rights and fundamental freedoms, including the right to development, gender equity, child welfare, youth interests, social inclusion, and the strengthening of civil society. Of relevance to women's economic empowerment (WEE)—and its impact on both women and their children—is the Charter's call to empower women and youth. It recognizes that building their capacities is not only essential for individual growth but also a critical driver of development and a valuable resource for the region's progress.

Under Article V on Employment and Working Conditions, the Charter calls on Member States to uphold every individual's right to work. This includes supporting access to financial resources for starting and growing small and medium-sized enterprises, as well as ensuring the provision of both maternity and paternity leave. Article VI on Labor Laws requires that Member States strive to achieve specifications of minimum rest periods, annual paid leave, compassionate leave, paid maternity leave, occupational health, and safety protection as one of the minimum standards in their labor legislation.

Article IX on the Wellbeing of the child and the most relevant to child care expectations that Member States shall strive to ensure include:

- Governments and relevant stakeholders should prioritize the holistic wellbeing of every child by ensuring they are nurtured in an environment that promotes their mental, physical, spiritual, and cultural development. This growth should occur under conditions that uphold their freedom, dignity, and overall health.
- Children must be safeguarded from all forms of abuse and exploitation that could harm their wellbeing. In every decision or action involving a child, their best interests and welfare should always come first and serve as the primary guiding principle.
- During their early years, children should ideally remain with their biological parents unless exceptional circumstances require otherwise. Special attention and support must be provided to those who lack family care or sufficient resources, with state assistance available to ensure their proper upbringing and maintenance.
- Efforts should also focus on enhancing essential services that support a child's development. These include quality pre- and postnatal care, early childhood education, nutrition programs, and immunization, all aimed at lowering rates of infant and child illness and death.
- Additionally, children facing legal challenges or those in vulnerable situations should receive effective rehabilitation and reintegration support. This includes access to education, healthcare, and safe living conditions.
- Children with special needs deserve tailored services that support their unique circumstances, and there should be continuous encouragement of active parental involvement in their care and development.

### The Economic Community of Central Africa States

The Economic Community of Central African States (ECCAS) (31) is primarily focused on fostering harmonious cooperation and promoting sustainable, balanced development across all areas of economic and social life in Central Africa. While ECCAS doesn't have information specifically related to ECC, it has taken meaningful steps toward advancing broader regional goals. In 2015, ECCAS entered a strategic partnership with UNICEF aimed at supporting the survival and development of children throughout Central Africa. This collaboration brought together ECCAS's dedication to regional peace, prosperity, and unity with UNICEF's deep expertise in child rights and welfare. Through this alliance, the two organizations worked together to champion children's rights, shaping policies and legislation that seek to enhance the wellbeing of both children and women in a region increasingly affected by instability and vulnerability. A key aspect of their cooperation included the joint creation and execution of resource mobilization strategies to secure funding for their shared initiatives.

#### The East African Community

The East African Community (EAC) (38) Treaty recognizes the important role of women and children in society and emphasizes the importance of protecting their rights. Yet, despite representing nearly half of the population, women continue to face marginalization, particularly in decision-making spaces. Compared to men, they often have less access to education, financial resources, and modern technologies such as information and communication tools. In response to these persistent disparities, the Partner States have made various efforts to promote gender equity. These efforts include the development and enforcement of policies and legal frameworks aimed at addressing the historical inequalities women have endured.

EAC draws its commitment to the rights and welfare of children from Article 120 of its founding Treaty. This article emphasizes the importance of collaboration among Partner States in matters of social welfare, particularly in supporting vulnerable and marginalized groups, including children. In response to this mandate, the EAC has established both Child and Youth Policies designed to serve as a regional framework for developing, coordinating, and enhancing initiatives that safeguard the rights and wellbeing of children and young people.

These policies are supported by detailed Action Plans, which outline key priority areas. These include the delivery of care and essential services to the most vulnerable, the reinforcement of national child protection systems, and the strengthening of community-level mechanisms. Additionally, the policies promote a comprehensive approach to quality education, healthcare, and social protection across the region.

Regarding women's empowerment, the EAC (38) Treaty—particularly Articles 121 and 122—acknowledges the vital contribution women bring to the socio-economic and political development of East Africa. Women contribute significantly through their roles in production, family health, early childhood education, and the preservation of cultural values. Moreover, Article 5(e) of the Treaty underlines the importance of integrating gender perspectives into all EAC activities, reinforcing the commitment to gender equality and inclusive development across the Partner States.

The East African Community (EAC) (38) Treaty, particularly Article 120(c), urges Partner States to take a unified stance in addressing the needs of marginalized populations, including children. This directive reflects the EAC's broader commitment to upholding key principles of good governance—such as democracy, the rule of law, accountability, transparency, social justice, equal opportunity, and gender equality. Additionally, it emphasizes the importance of promoting and protecting human and peoples' rights, in line with the African Charter on Human and Peoples' Rights [Article 6(d)].

Individually, EAC Partner States have taken steps by ratifying various international and regional instruments on children's rights and implementing corresponding legal, policy, and programmatic measures. Despite these national efforts, a coordinated regional strategy for enforcing children's rights has been lacking. The 2016 EAC Child Policy represents a significant milestone toward harmonizing regional standards and approaches to child rights within the Community. Its development was informed by key regional initiatives, including the 2012 Bujumbura Declaration on Child Rights and a directive from the 25th Council of Ministers Meeting, which tasked the EAC Secretariat with creating a comprehensive policy on children's rights and wellbeing (Directive 23, EAC/CM 25).

Supported by the Inter-Agency Working Group, the EAC Secretariat launched the policy development process in 2013. The policy was designed to: (a) Establish a regional framework to support and align national efforts in advancing children's rights and wellbeing; (b) Help Partner States integrate child rights priorities into planning, budgeting, and resource allocation; (c) Enhance cross-border collaboration on child protection and welfare; (d) Support the monitoring and evaluation of the implementation of the UN Convention on the Rights of the Child (UNCRC), the African Charter on the Rights and Welfare of the Child (ACRWC), as well as related national obligations; (e) Strengthen research, advocacy, and innovation in the field of child rights across the region.

### National/subnational policy directions on ECC (case study of Kenya)

In the Kenyan context, care for children in the early years traverses the health and education sectors. It turns out that this is a disadvantage as neither sector considers child care as their primary mandate. The education sector focuses on primary education and 1–2 years of pre-primary schooling, with little attention to the earlier years, while the health sector focuses its services on preventive and curative services. Consequently, there is considerable urgency to find effective ways that are informed by government policies to improve child care. Since the enactment of Kenya's 2010 Constitution, which devolved the ECD sector to county governments, the sector has not received the attention it deserves. The Constitution introduced a two-tier system of governance, national and county governments that are both distinct and interdependent. This shift calls for a new approach to integrated development planning, one that unites the various aspects of development under a coordinated framework. Achieving this vision relies heavily on strong collaboration between the national and county governments, as well as with other key stakeholders, to effectively address the evolving needs of society. In reviewing policy frameworks related to child care, we found a range of national policy documents alongside County Integrated Development Plans (CIDPs) from all 47 counties. These were analyzed with a focus on the nurturing care framework and variable strengths, gaps and areas for improvement were revealed.

#### National policies

The Children's Act of 2001, as revised in 2022, is overseen by Kenya's Ministry of Public Service, Gender, Senior Citizens Affairs, and Special Programmes. This legislation outlines key areas related to the welfare of children, including guardianship, adoption, fostering, maintenance, parental responsibility, and overall care and protection. While the Act doesn't explicitly reference early childhood care (ECC), it strongly emphasizes the rights of the child under Part II. It obliges the government to utilize the fullest extent of its available resources to progressively realize these rights. Among these is a child's inherent right to life, with both the government and the family bearing the responsibility for ensuring the child's survival and holistic development—always acting in the best interests of the child. Additionally, the Act affirms that every child has the right to live with and be cared for by their parents, as well as access to healthcare, which is a shared responsibility between parents and the state. When it comes to protection, the Act ensures children are safeguarded from physical or psychological harm, neglect, and any form of exploitation.

Part III of the Act emphasizes the essential role of parents in ensuring a child's wellbeing. This includes meeting the child's basic needs such as proper nutrition, safe shelter, clothing, healthcare, education, and emotional guidance. When parents are married, this responsibility is shared equally between them. However, in cases where the parents are not married, the primary responsibility typically falls to the mother. Additionally, the National Council for Children's Services plays a central role in overseeing child welfare efforts—this includes planning, funding, and coordinating the various initiatives outlined in the Act.

The Children Act of 2022, which replaces the earlier 2001 legislation, introduces several important updates and improvements. Notably, it expands definitions to address contemporary issues such as online abuse, child exploitation, and radicalization. The Act also establishes a Child Welfare Fund, supported by the national treasury, and explicitly directs county governments to develop child welfare programs and child care services. Additionally, the law mandates free medical treatment, specialized care, education, and training for children with disabilities. It places greater emphasis on family-based alternative care over institutionalization, recognizing the critical importance of parental care. To support this, the Act includes a range of alternative care options such as kinship care, guardianship, adoption, kafaalah, foster care, emergency care, temporary shelter, supported independent living, and assistance for child-headed households.

The National Early Childhood Development (ECD) Framework, introduced in 2006 (28), aims to support all children from conception through the age of eight—including those who are vulnerable or marginalized. The framework is operationalized through the Early Childhood Development Service Standard Guidelines, which specify requirements for teacher qualifications, infrastructure (safe physical environment, sanitation, and learning materials), and integrated services that go beyond just providing education to include health screenings and nutrition programs.

The framework clearly outlines the shared responsibilities of various players involved in delivering ECD services. These partners range from parents and caregivers to community members, relevant government ministries (such as education, health, water, and local government), as well as universities, research institutions, NGOs, community-based organizations, and development partners at both the bilateral and multilateral levels.

A few key commitments stand out in this policy framework. First, the government, working closely with its partners, is expected to develop and implement strategies that make training in early childhood service delivery widely accessible, with a focus on ensuring fair distribution of opportunities, especially in underserved areas. Second, the framework calls for the creation and monitoring of clear, user-friendly standards and quality guidelines that apply across sectors and settings. These guidelines aim to protect and advocate for children's rights and are to be reviewed periodically as needed. Third, the government pledges to boost financial support for programs targeting young children and their families, to enhance quality, access, and equitable service provision, particularly for children with special needs or those in disadvantaged communities.

In addition, the framework aligns itself with key international and regional commitments, including the UN Convention on the Rights of the Child (CRC), the Sustainable Development Goals (SDGs), and the African Charter on the Rights and Welfare of the Child (ACRWC). It also recommends that the National Council for Children's Services establish a coordinating committee to integrate sectorial efforts and ensure that donor contributions are aligned and effective.

Despite its comprehensive nature, the National ECD Framework implementation remains a challenge due to financing gaps and the heavy reliance on donors and inconsistent government resource allocation. This is compounded by the limited teacher capacity, with about half of the teachers untrained and not up to date with child-centered play-based methods, as well as weak data integration between national and county-level authorities.

The Care and Protection of Child Parents Bill (2019) provides a framework for the care and protection of child parents while at the same time ensuring optimum care for their children, and of standards for the establishment and regulation of care centers by county governments. Under this Bill, each County Executive Committee Member is expected to put in place management plans and strategies for the delivery of social services and child care support services to expectant children and child mothers. The Bill is specific that the childcare centers are for children below the age of 3 years, and requires that the services offered should be affordable, accessible including to children with special needs and of good quality. The Bill also highlights the need to promote and protect the welfare of the children within the centers. The National Council for Children's Services (NCCS) is mandated to put in place mechanisms that will help establish a comprehensive capacity building program for child parents to ensure they practice responsible family life. NCCS is also mandated to establish non-discriminatory enrolment back to school or other training programs as well as identify children who are pregnant and in need of interventions to enable them realize their rights. It is also NCCS' function to address any educational and related barriers, and, guarantee funding and sustainability of initiatives to support child parents. The bill is aimed to safeguard expectant children and teen parents by guaranteeing their right to education and establishing county-run, regulated care centers for their children. It compels governments to curb school dropouts, provide support services, and ensure proper care.

The bill provides for school re-entry particularly adoption of a non-discriminatory approach to pregnant students and provide them with guidance and support for a smooth return after delivery. The Bill requires that all cases of student pregnancy must be handled professionally and confidentially by school principals to protect the child parent from stigma. It provides that the childcare centers are licensed and inspected to ensure that they meet the health and safety requirements for the infants. Ultimately, the main goal of the bill and its provisions is to balance education continuity with the care of children born to minors. However, there have been criticism that these provisions are already partially covered by the Children Act 2022, highlighting a duplication between these two frameworks. Furthermore, as with other frameworks, the bill suffers from implementation gaps due to limited financing and potential resistance as it has sometimes been perceived to inadvertently normalize or promote teenage pregnancy.

#### County Childcare Facilities Acts

The Nairobi City County Childcare Facilities Act of 2017 was established to guide the registration, licensing, and regular evaluation of childcare centers within Nairobi County. Its main goal is to create a legal structure that ensures children in these facilities are well protected and cared for, aligning with the principles outlined in the Children's Act. This Act makes it possible for working parents or those temporarily unable to care for their children to leave them in environments that are safe, nurturing, and conducive to early learning. Oversight of these facilities falls under the Department for Education, Youth Affairs, Culture, Children, and Social Services. This department is responsible for regulating and licensing childcare centers, monitoring their adherence to quality and safety standards, developing relevant policies and operational standards, addressing complaints, and ensuring alignment with directives from the County Education Board. Additionally, they oversee the implementation of early childhood curricula and carry out any other necessary functions related to childcare services.

Other counties that have provisions for the management and licensing of childcare facilities include Embu (21), Mombasa (22), Mandera (19), Nakuru (20), and Tana River (23). Whereas some of the Acts are specific to childcare facilities, others (e.g., the Mandera Act) apply to all orphanages and childcare facilities within the county. One of the recommendations in the implementation of these Acts is to ensure that child care activities within the counties conform to the national and county legislations on health and safety of young children. The Acts cover all NCF components and address all children under 6 years although their implementation remains a challenge. Moreover, the standards outlined in the document are out of the reach of many childcare facilities, particularly those in informal settlement and other marginalized settings as was revealed by a recent study in Nairobi (24).

#### County integrated development plans

Overall, all CIDPs recognize the need for childcare centers. However, as is highlighted in an earlier publication, the CIDPs do not specify the age range of the children targeted by these centers and none of them caters for the care of children below 3 years (25). The needs of children in the 4–5-year age group were mentioned frequently, and this translated into the budgets presented within the CIDPs, particularly in relation to the building and staffing of ECDE centers for this age group (25). The CIDPs place a lot of emphasis on good health, adequate nutrition, security and safety as well as water, sanitation, and hygiene (WASH). However, the responsive caregiving and opportunities for early learning components receive limited coverage and rarely have any budget allocation (25).

While frameworks such as the Nairobi Childcare Act of 2017 (26) are in place to support young children, their translation into actionable and well-funded plans remains inconsistent. As urbanization accelerates and more women join the workforce, there's an urgent need for policies to keep pace with the shifting realities of families—particularly in relation to childcare centers. This includes recognizing the role of informal childcare settings and addressing the needs of children under the age of four. To effectively promote early childhood development, policies must outline clear minimum standards, and implementation plans should be backed by resources tagged to child care needs across the aspects of the nurturing care framework. Blanket budgets and plans that do not explicitly commit funds to child care mean that it will continue to lag behind other priorities.

## Discussion

The current review examined the early care policy landscape globally, in Africa and in Kenya to identify the strengths, roles of different stakeholders, challenges in implementation, and key gaps that need to be addressed. Several policies operating at different levels, to guide the provision of different aspects of early child care and addressing respective domains of nurturing care are discussed. Global policies generally focus on high-level policy thrusts aimed at ensuring that the environment in which children live allows them to thrive and achieve their full potential. Additionally, global policies also occasionally address specific issues of ECC. For instance, the UN 2019 Resolution, and the World Bank Agenda on Gender seek to address the challenges associated with child care and women living in diverse circumstances to protect children rights and wellbeing, as well as ensure equitable participation of women in the economic development of society. The UN 2019 Resolution also speaks to the issue of children without parental care highlighting the right of the child to grow up in a family environment and the rights of children with disabilities.

The global policy frameworks provide guidance to ensure the healthy development and wellbeing of all children. These high-level policies serve as a reference for regional and national policies and guidelines that will enable implementation toward achieving the global targets. For example, while the SDG 4 emphasizes the need for responsive care and parenting for early learning, and highlights care areas that should be addressed. These should be specified in the national policies and subnational guidelines for implementation.

The ILO specifically recognizes the gender-based inequalities in child care with women are at the disadvantage. ILO member states resolution at the ILO international Conference in 2024 was timely. This resolution to promote descent work and care economy and championing the effort to address child care challenges and harness the opportunities it presents. The value of well-functioning care economy extends beyond individuals and families to the promoting healthier workforce. ILO illuminates the structure barriers to women participation in paid work that are associated with unpaid care work, with about 708 million women globally unable to engage in the labor market. https://www.ilo.org/resource/news/unpaid-care-work-prevents-708-million-women-participating-labour-market.

Effective implementation of global policy targets for child care requires that the policies are cascaded downward and customized through the regional and local policies. This review revealed vertical linkages between the policy frameworks, at the different levels, and across institutions at the same level. For example, the African Development Bank's agenda borrows heavily from the global SDGs and collaborating with institutions through the Banking on Nutrition Partnership and the Gray Matter Infrastructure to promote full physical and cognitive potential of children in Africa toward long-term impacts on health outcomes and economic development of the continent.

Similarly, the AU CESA addresses the roles of different stakeholders in the care of young children citing the critical contribution of global institutions like the AfDB, WB, DfID, and EU in terms of providing financing for child care. While addressing the role of caregivers, the AU CESA recognizes the need to ensure that children in special circumstances are well taken care of and acknowledges that such families may need additional support to help their children thrive. This and other instruments recognize the need for states to develop and implement progressive social policies and practices to address current challenges in child care. Here again the role of interlinkages between different levels of actors in supporting child care policies is demonstrated.

At the national level, even though ECD became a devolved function in Kenya (since 2010), there is still a strong connection between county guidelines and national policies as county governments are expected to align their Acts, CIDPs and implementation plans with the National ECD and child care policies. However, the review revealed that national policies (ECD policy) were not clear on early child care. The Nairobi Childcare Facilities Act provides specific guidelines; however, their implementation is crippled by lack of translation into achievable plans, and limited budget allocation. Moreover, the stipulated standards are far from what can be achieved by care providers in the poorest communities. This clarity gap is also reflected in the CIDPs as they also lack clear plans and budgets specific to child care. Of note, responsive caregiving and opportunities for early learning components received limited coverage in the CIDPs and rarely had any budget allocation.

Increasingly there is a recognition of the early years and the critical role they play in the developing human being. The global, national, and subnational laws and policies address the different aspects of ECC. Formal care services, however, seem to receive more focus in the legal and policy frameworks while informal care options, particularly within the child's immediate environment, remain largely ignored, yet these need more support. Across the different policy instruments, there is a consistent focus on health and education, and apart from the WHO's NCF, child care is hardly mentioned in the other instruments. Although the policies offer guidance and recommendations, implementation frameworks, mandates and budgets are largely missing. Further, at the regional, national and subnational levels, none of the policies and frameworks have outlined indicators to measure progress. Moreover, there is not much adaptation of the success indicators (such as the SDGs) to the African context and the facilitating role of African institutions remains largely underutilized.

Concerning targeted populations, many of the policy instruments are not explicit about provisions for children aged below 3 years. For instance, the needs of young children have not been comprehensively addressed in Poverty Eradication Strategy Papers (which could also be considered as implementation plans). About the rights and welfare of children, most of the instruments recognize this as being important. However, some of them such as The Children's Act place a lot of emphasis on institutionalized children while being silent on issues around care of children within the home environment. Although the policy instruments call for multi-sectorial coordination across health, nutrition, agriculture, education, and child protection, much remains to be done. The African Union highlights the vital role that parents and caregivers play as a child's first educators. It also underscores the importance of strong advocacy efforts to drive political commitment and leadership, while calling for increased support in developing, implementing, monitoring, and evaluating effective policies.

The 0–3-year gap is also evident in both national policies and CIDPs where care for children under 3 years is not mentioned. It is assumed that children at that age are always at home and being cared for by their families, yet increasingly children are taken to child care facilities as early as 6 months.

In a similar vein, policies provide guidance for parenting but because they do not specify roles of caregiving, it leaves the job to the women (mothers) as the default caregivers and therefore allowing the unequal distribution of the burden of child care to the women. For example, the WHO NCF is explicit on the key aspects that must be provided for children. However, the NCF does not provide specific information on who (carers) should do what for the child. Without specifying the roles the different caregivers, it assumes the default status where mothers carry the entire burden. While the role of fathers in child care is increasingly gaining more recognition, child care guidelines and policy frameworks remain vague on this. Similarly, the UN Convention for Children's rights is also silent about the role of each parent (or caregiver).

SDG 4 emphasizes the need for responsive care and parenting for early learning, and highlights care areas but is also not specific on who is responsible for specific roles. Specificity in regards to who should provide child care is important as it recognizes the unequal distribution of child care, and therefore the need for the regulatory frameworks promote equitable distribution of the burden of child care between two parents. The deliberate shift to clarify child care roles should go beyond just articulation in the policies and guidelines to promoting awareness and mindset shifts especially in contexts where care roles are still largely driven by gendered norms.

Further, the development of costed implementation plans will enable governments to pinpoint funding gaps and will facilitate identification of needs and mechanisms for action tagged to child care. Moreover, identification of a lead agency or department to spearhead coordination and implementation is critical. The lead agency needs to be well staffed, adequately financed, and capable of managing communication and collaboration across several departments.

Policies and legal frameworks that advocate for vulnerable children e.g., Southern African Development Community (SADC) do not mention ECC, and there is no age category indicated for orphans and vulnerable children rendering the policy hard to enforce or implement. This assumes that all children irrespective of age have relatively similar needs; even though, children 0–3 have unique needs which make them more vulnerable than older children.

Overall, the current policy landscape underpins the key areas of nurturing care for children, and so much has been achieved. However, a lot is yet to be done toward effective implementation for greater impact as highlighted in the following recommendations. Policy documents need to be more explicit on what is expected in terms of quality of care, who must provide care (father and mother), and outside the household, what are the acceptable options for child care. There is need to foster or strengthen linkages between policy frameworks and the different players to enable cascading of goals and effective coordination on implementation, There is need for clarity in the roles of the different players including the role of fathers in care, and address the disproportionate burden of early child care on women. It is important to provide the necessary support and resources including funds to enable implementation of the policies especially in economically disadvantaged communities where the current guidelines for child care are almost unachievable without external support. It is also crucial to have a system in place to regularly track and evaluate policy implementation and impact against the intended outcome indicators for accountability and to maximize impact. It is evident, and as highlighted by the AU, that there is need for commitment, advocacy and joint effort at all levels of decision making to ensure that child care issues are loudly articulated and addressed within the different policy frameworks. Lastly, it is important to have a systems thinking approach to early childcare, and shift from fragmented policy implementation toward an integrated childcare ecosystem that fosters synergy and leveraging of resources for amplified child outcomes.

### Limitations

We recognize that it might be difficult to identify all the challenges in implementation through just a policy review. Engagement of stakeholders would have provided more information on the different aspects of the policies where such information might be missing in the documents. Within the main project, the plan was to conduct interviews and discussions with stakeholders for their insights on the strengths and weaknesses of the policies as well as suggestions for improvement. Further, we also recognize that in much as countries in sub-Saharan Africa may be similar in ECC policy frameworks since these are drawn from the regional frameworks, specific aspects such as the subnational legal frameworks (CIDPs) and implementation approaches are unique to the Kenya context and may not be generalizable to other contexts. Lastly, this paper is based on the review which was 4 years ago prior to the publication of the article. We recognize that since then, more literature might be available; new policies emerged or changes to some of the policies that were reviewed may have occurred.

### Implications for future research and policy advocacy

Future policy evaluations should include both desk-base review as well as stakeholder engagement to obtain deeper insights and more information that may not be exhaustive from the documents. Moreover, studies should include wider scope in terms of countries, since findings from a single country may not necessarily be generalizable to the rest of the continent. Nevertheless, this policy review highlights pertinent aspects that can guide policy development processes, implementation and advocacy frameworks not only for child care but also for any issue at hand. Effective or successful policies should be more explicit, and have clear implementation frameworks including the resources required and an evaluation plan to track impact. Ideally, a comprehensive evaluation of policy frameworks including their success and impact would require comprehensive and longitudinal implementation research.
